# Remodeling of the cycling transcriptome of the oyster *Crassostrea gigas* by the harmful algae *Alexandrium minutum*

**DOI:** 10.1038/s41598-017-03797-4

**Published:** 2017-06-14

**Authors:** Laura Payton, Mickael Perrigault, Claire Hoede, Jean-Charles Massabuau, Mohamedou Sow, Arnaud Huvet, Floriane Boullot, Caroline Fabioux, Hélène Hegaret, Damien Tran

**Affiliations:** 1 University of Bordeaux, EPOC, UMR 5805, F-33120 Arcachon, France; 2 CNRS, EPOC, UMR 5805, F-33120 Arcachon, France; 3 Plate-forme bio-informatique Genotoul, MIAT, Université de Toulouse, INRA, F-31326 Castanet-Tolosan, France; 4 Ifremer, Laboratoire des Sciences de l’Environnement Marin (LEMAR), UMR 6539 UBO/CNRS/IRD/IFREMER), CS 10070, F-29280 Plouzané, France; 5 Laboratoire des Sciences de l’Environnement Marin (LEMAR), Institut Universitaire Européen de la Mer, Université de Bretagne Occidentale, UMR 6539 CNRS/UBO/IRD/IFREMER, F-29280 Plouzané, France

## Abstract

As a marine organism, the oyster *Crassostrea gigas* inhabits a complex biotope governed by interactions between the moon and the sun cycles. We used next-generation sequencing to investigate temporal regulation of oysters under light/dark entrainment and the impact of harmful algal exposure. We found that ≈6% of the gills’ transcriptome exhibits circadian expression, characterized by a nocturnal and bimodal pattern. Surprisingly, a higher number of ultradian transcripts were also detected under solely circadian entrainment. The results showed that a bloom of *Alexandrium minutum* generated a remodeling of the bivalve’s temporal structure, characterized by a loss of oscillations, a genesis of de novo oscillating transcripts, and a switch in the period of oscillations. These findings provide unprecedented insights into the diurnal landscape of the oyster’s transcriptome and pleiotropic remodeling due to toxic algae exposure, revealing the intrinsic plasticity of the cycling transcriptome in oysters.

## Introduction

The physiological and behavioral processes of living organisms oscillate. Temporal organization is a necessary adaptation to cope with an ecosystem governed by various periodic changes^1^. At a cellular level, the circadian clock consists of a self-sustainable auto-regulatory network of transcriptional and translational feedback loops synchronized by environmental zeitgeber, which produce oscillations in the expression of clock-controlled genes (CCGs)^2^. Most studies on the circadian network have used terrestrial organisms, while little is known about these networks in marine organisms, which inhabit complex ecosystems governed by sun-earth-moon trajectories and interactions. The study of clocks in marine species is a relevant issue for understanding how clocks evolved along different evolutionary lineages^3–10^.

In the phylum of marine molluscan, the Pacific oyster *Crassostrea gigas*, an ecologically and economically important marine species, exhibits *in situ* behavior following not only a circadian rhythm but also ultradian and infradian rhythms^11, 12^. An endogenous circadian rhythm has been demonstrated in free running conditions and is characterized as weak, plastic, and dual^13, 14^. Recently, components of a biological clock in *C. gigas* have been identified^15^ without any information on the extent of clock output^15^. The first study in marine mollusks showed that 40% of microarray transcripts are susceptible to oscillation in the mussel *Mytilus californianus* ^3^. In mammals, next-generation sequencing has indicated that approximately 50% of the genome has the potential to oscillate; based on a sophisticated regulation of gene expression in each peripheral organ, which is still misunderstood, this estimation has been reduced to 10% in a given tissue^16–22^. In parallel, alterations in the temporal organization of physiological and metabolic processes have been associated with disorders such as obesity, cardiac disease, cancer or disruption of energy balance^23–29^.

In coastal areas, marine organisms are subjected to various stresses, including proliferations of planktonic microalgae producing toxins, called harmful algal blooms (HABs), reaching up to millions of cells per liter. Among these planktonic algae, the dinoflagellate species *Alexandrium minutum* is known to produce saxitoxin (STX) and its derivatives, called paralytic shellfish toxins (PSTs). *A. minutum* has a worldwide distribution and has been associated with massive deaths of marine animals and human health issues^30–32^. The oyster *C. gigas*, as a filter feeder, is highly susceptible to HAB exposure by feeding on harmful algae^33^. Much physiological damage in response to *A. minutum* exposure has already been demonstrated in oysters^14, 34–39^. In particular, it has been shown that *A. minutum* leads to a loss of daily cycle in the expression of genes involved in detoxification, oxidative stress, valve behavior, and crystalline style length, as implicated in digestion^40^.

In the present study, we used RNA sequencing (RNAseq) to investigate for the first time, to our knowledge, (1) the manner in which the whole transcriptome in gills of *C. gigas* is temporally organized under light/dark entrainment and (2) the impact on this temporal organization of an *A. minutum* exposure mimicking a realistic HAB.

## Results

### A circadian transcriptome with a nocturnal pattern remodeled by *A. minutum* exposure

To assess the cycling transcriptome of *Crassostrea gigas*, a first group of oysters was entrained to a 9-hr light/15-hr dark (L: D 9:15) environment, without tidal cycles, and fed with the non-toxic alga *Heterocapsa triquetra* (*H.t* condition). To explore the effect of PST ingestion, a second group of oysters was exposed to the PST-producing alga *Alexandrium minutum* (*A.m* condition), mimicking a harmful algal bloom (≈400 cells.mL^−1^). ELISA assays confirmed a PST bioaccumulation of 95.8 ± 13.5 ng.g^−1^ eqSTX in the gills in the *A.m* group, whereas the *H.t* oysters accumulated no PST (Supplementary Fig. S1). Gills were sampled every 4 hr over 52 hr, and the cyclic transcriptome was analyzed by RNA sequencing (RNAseq) followed by ARSER^41^ to evaluate any significant cyclicity in the 20,846 transcripts. For all cycling detection, a false discovery rate (FDR) of <0.05 was considered significant^42, 43^.

**Figure 1:**
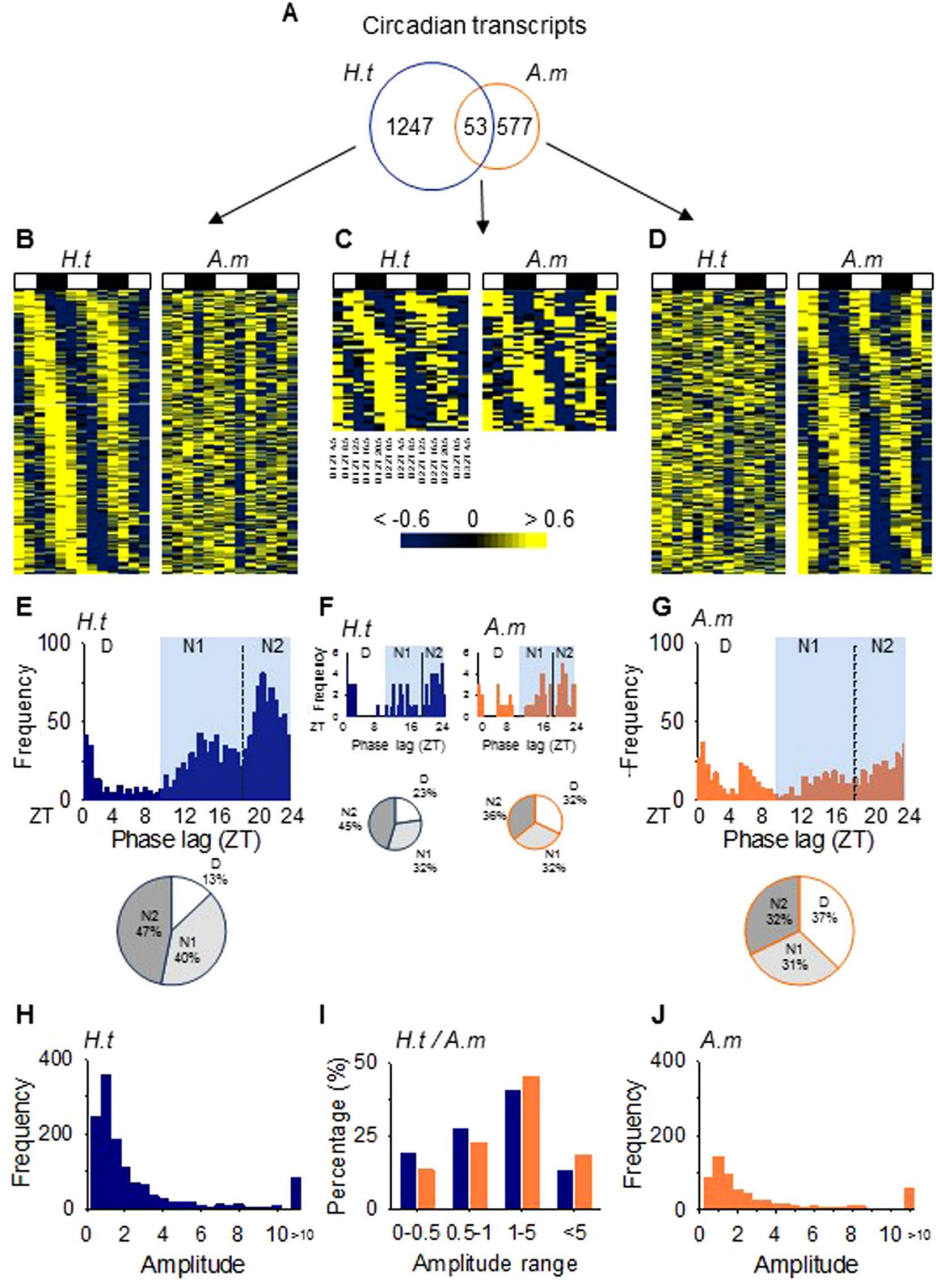
A circadian transcriptome with a nocturnal pattern remodeled with *A. minutum* exposure. (**A**) The number of circadian transcripts. (**B**–**D**) Heat maps of expression organized by phase for the 300 most significant transcripts oscillating only in *H.t* (**B**), common in both (**C**) and only in *A.m* (**D**). (**E**–**G**) Phase lag, i.e., peak of expression along the cycle, distributions (frequency in number of transcripts) and corresponding pie charts for all oscillating genes in *H.t* (**E**), *A.m* (**G**) and both (**F**). “D”: phase lag distribution during the day (ZT0-ZT9). “N1” (ZT9-ZT19) and “N2” (ZT19-ZT24): phase lag distribution during the first and second part of the night. (**H**–**J**) Amplitude distribution (frequency in number of transcripts) for all circadian transcripts in *H.t* (**H**), *A.m* (**J**), and both (**I**), classed by amplitude range.

In the *H.t* oysters, 1300 (1247 + 53) significant circadian transcripts were identified, corresponding to 6.2% of total transcripts in the *H.t* gills (Fig. 1A–C; Supplementary Table S2). Among these 1300 transcripts, only 53 oscillated in both the *H.t* and *A.m* oysters (Fig. 1A,C). The total number of circadian transcripts in the *A.m* group (630) was two-fold lower than that in the *H.t* oysters (Supplementary Table S2). A large number of transcripts (577) oscillated exclusively in *A.m* (Fig. 1A,D). In *H.t*, the phase lag distribution of circadian transcripts showed a nocturnal and bimodal pattern, with one peak during the beginning of the night (N1, ZT9-ZT19, 40% of circadian transcripts) and another peak at the end of the night (N2, ZT19-ZT24, 47% of circadian transcripts) (Fig. 1E). Finally, only 13% of transcripts peaked during the daytime (D, ZT0-ZT9). In *A.m*, the nocturnal phase peak transcripts decreased to 63% (N1+N2), and diurnal transcripts reached 37% of circadian transcripts (Fig. 1G). An increase in daytime peak expression with *A. minutum* exposure was also observed in the 53 circadian transcripts common in both *H.t* and *A.m* (Fig. 1F). Furthermore, we observed a trend toward a relative decrease in significant transcripts with low amplitude in favor of transcripts oscillating with high amplitude when exposed to *A.m* compared to *H.t* (Fig. 1H–J).

### A nocturnal pattern of circadian core clock gene expression and valve activity behavior modeled with *A. minutum* exposure

**Figure 2:**
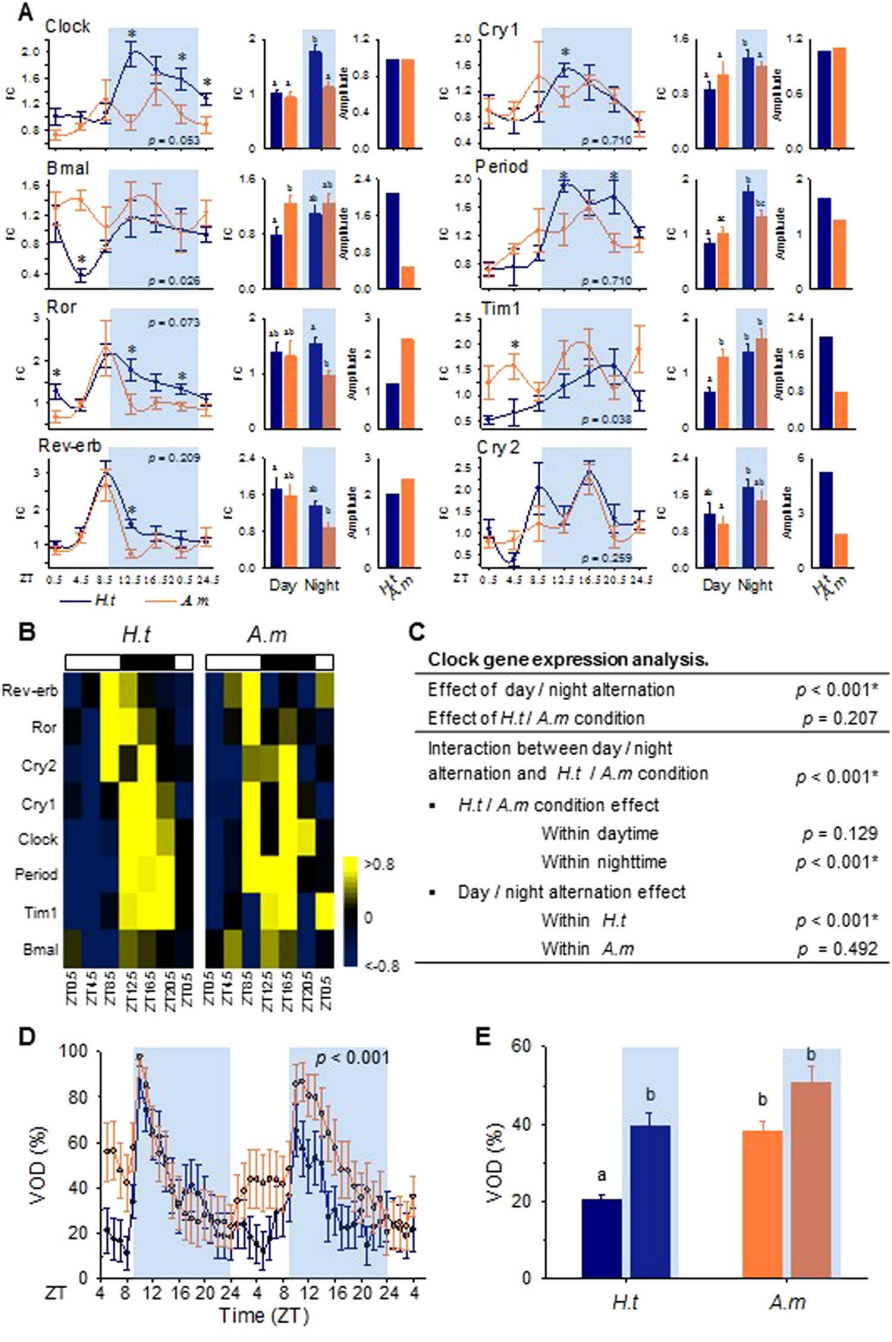
A nocturnal pattern of circadian core clock gene expression and valve activity behavior modeled by *A. minutum* exposure. (**A**) Real-time qPCR analysis of circadian clock gene expression expressed in fold change (FC, mean ± SE, n = 5–6) for *H.t* and *A.m*. Gray areas correspond to nighttime. The *p*-values show different levels of expression over 28 hr between *H.t* and *A.m*, while stars indicate the different levels of expression at each time point. The first histograms show the mean levels of gene expression (FC). Different letters correspond to significant differences (*p* < 0.05). The second histograms show amplitudes of clock gene expression in *H.t* and *A.m*. (**B**) Heat maps of clock gene expression over 28 hr, organized by phase for *H.t* and *A.m* (RT-qPCR). (**C**) Two-way ANOVA testing the effect of day/night and *H.t*/*A.m* conditions on overall clock gene expression. (**D**) Mean hourly valve opening duration (VOD, %; mean ± SE; n = 16 per condition) of *H.t* (blue) and *A.m* (orange) oysters during the two days of sampling. The *p*-value corresponds to the difference in the mean VOD between the two conditions. (**E**) Mean VOD during daytime and nighttime for each condition. Different letters indicate significant differences (*p* < 0.05).

Oyster clock gene expression in the gills of oysters was analyzed by RT-qPCR and exhibited nocturnal phase peaks in *H.t* (Fig. 2A,B). There were significant differences between day and night phase expression for *Clock*, *Cryptochrome 1 (Cry1)*, *Period* and *Timeless (Tim1)*, but not for *Ror*, *Rev-erb*, *Cryptochrome 2 (Cry2)* or *Bmal*, peaking just before the night or at the beginning of the day (Fig. 2A, *H.t*, first column of histograms). Fig. 2A and B reveal an effect of *A. minutum* on the clock gene temporal patterns previously observed in *H.t*. First, the overall levels of expression were higher in *A.m* for *Bmal* (*p* = 0.026) and *Tim1* (*p* = 0.038) (Fig. 2A). In addition, none of the 8 clock genes showed differential expression between day and night in *A.m* (Fig. 2A, *A.m*, first column of histograms), leading to a loss of nocturnality. Furthermore, in *A.m*, the amplitude of oscillation decreased for *Bmal*, *Period*, *Tim1* and *Cry2* and increased for *Ror* and *Rev-erb* (Fig. 2A, second column of histograms). A two-way ANOVA confirmed a day/night effect on clock gene expression in *H.t* (*p* < 0.001) but not in *A.m* (*p* = 0.492) and a significant effect of *A. minutum* on gene expression during the night (*p* < 0.001) (Fig. 2C). Finally, significant correlation (*p*-value < 0.0001 for both conditions) between qPCR and RNAseq results on global clock gene expression in *H.t* and *A.m* conditions, comforting results of RNAseq analyses (Supplementary Fig. S2).

Figure 2D shows the mean valve opening duration (VOD) in the *H.t* and *A.m* oysters. Control oysters exhibited cycling and nocturnal valve activity with a rapid increase in VOD at the beginning of the night, corresponding to the N1 phase illustrated in Fig. 1E. This increase in VOD was followed by a decrease to basal levels, observed during the rest of the night and during daytime. The *A.m* oysters exhibited conserved cyclic valve activity, but the mean VOD was significantly increased (*p* < 0.001) and a trend for diurnal activity was observed corresponding to the D phase, shown in Fig. 1G. Figure 2E shows that during the day, the VOD increased from 20.6 ± 1.2% in *H.t* to 38.0 ± 2.6% in *A.m* (*p* < 0.001), whereas during the nighttime, no significant difference in VOD was observed (*p* = 0.305). Finally, the nocturnality observed in *H.t* was disrupted in *A.m*. Indeed, the day/night difference in *H.t* (*p* < 0.001) was abolished in *A.m* (*p* = 0.763).

### Discovery of an ultradian transcriptome remodeled by *A. minutum* exposure

**Figure 3:**
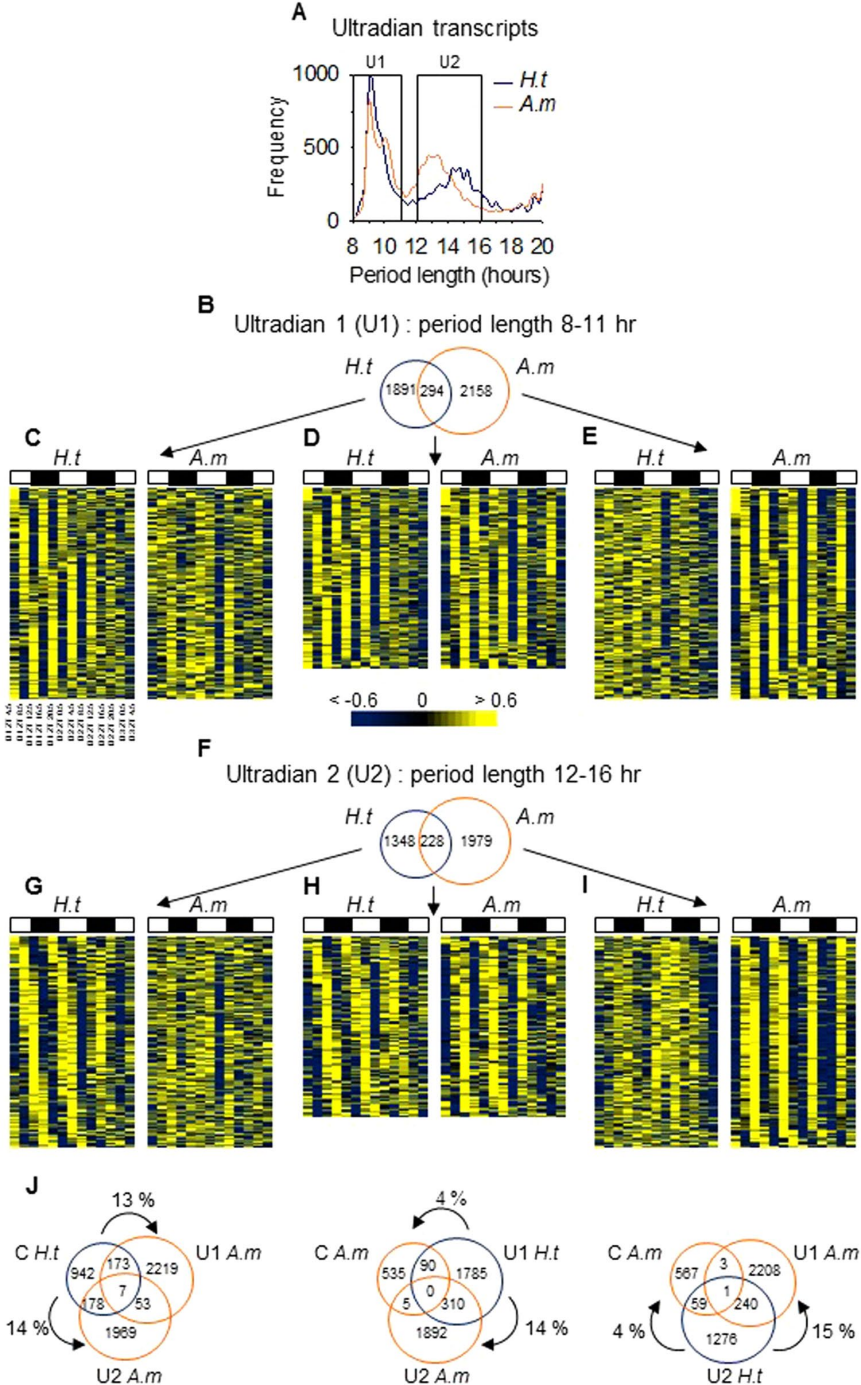
Discovery of an ultradian transcriptome remodeled with *A. minutum* exposure. (**A**) Period distribution of ultradian transcripts in *H.t* and *A.m*. (**B**) The number of U1 transcripts in *H.t* and *A.m*. (**C–E**) Heat maps of expression organized by phase for the 300 most significant U1 transcripts oscillating only in *H.t* (**C**), common in both (**D**), and only in *A.m* (**E**). (**F**) The number of U2 transcripts. (**G**–**I**) Heat maps of expression organized by phase for the 300 most significant U2 transcripts oscillating only in *H.t* (**G**), common in both (**H**), and only in *A.m* (**I**). (**J**) Venn diagrams of circadian (**C**), U1 and U2 transcripts in *H.t* and *A.m*.

Analysis of the cyclic transcriptome provided evidence for a large amount of transcripts with ultradian periodicity. Within this range, two major clusters of transcripts appeared in both *H.t* and *A.m* conditions (Fig. 3A): transcripts oscillating with a period in the range of 8–11 hr (Ultradian 1, U1) and transcripts oscillating with a period in the range of 12–16 hr (Ultradian 2, U2). For the U2 transcripts, the peak of period length distribution was shortened in *A.m* oysters. Ultradian transcripts (U1 + U2) represented 18.1% of the transcriptome in *H.t* and 22.4% in *A.m* (Supplementary Table S2). In *H.t*, 2185 (1891 + 294) transcripts were U1 (Fig. 3B–D) whereas in *A.m* oysters, 2452 (2158 + 294) transcripts were U1 (Fig. 3B,D,E), representing 10.5% and 11.8% of the transcriptome in *H.t* and *A.m*, respectively (Supplementary Table S2). Note, only 294 U1 transcripts were common to *H.t* and *A.m* (Fig. 3B,D).

Subsequently, in *H.t*, we observed 1576 (1348 + 228) U2 transcripts (Fig. 3F–H), whereas in *A.m*, 2207 (1979 + 228) transcripts were U2 (Fig. 3F,H,I), representing 7.6% and 10.6% of the transcriptome, respectively (Supplementary Table S2). Finally, only 228 U2 transcripts were common to *H.t* and *A.m* (Fig. 3F,H). A gene-to-gene comparison of cycling transcripts in *H.t* and *A.m* showed that *A. minutum* exposure led to a switch in the period range of many transcripts (Fig. 3J). Indeed, 13% and 14% of circadian transcripts in *H.t* became U1 and U2 in *A.m*. By contrast, 14% of U1 in *H.t* became U2 in *A.m*, while 15% of U2 in *H.t* became U1 in *A.m*. When exposed to *A. minutum*, the switch from an ultradian period to a circadian one was scarce, 4% for both U1 and U2 transcripts.

### Ultradian oscillations observed in circadian clock network elements

**Figure 4:**
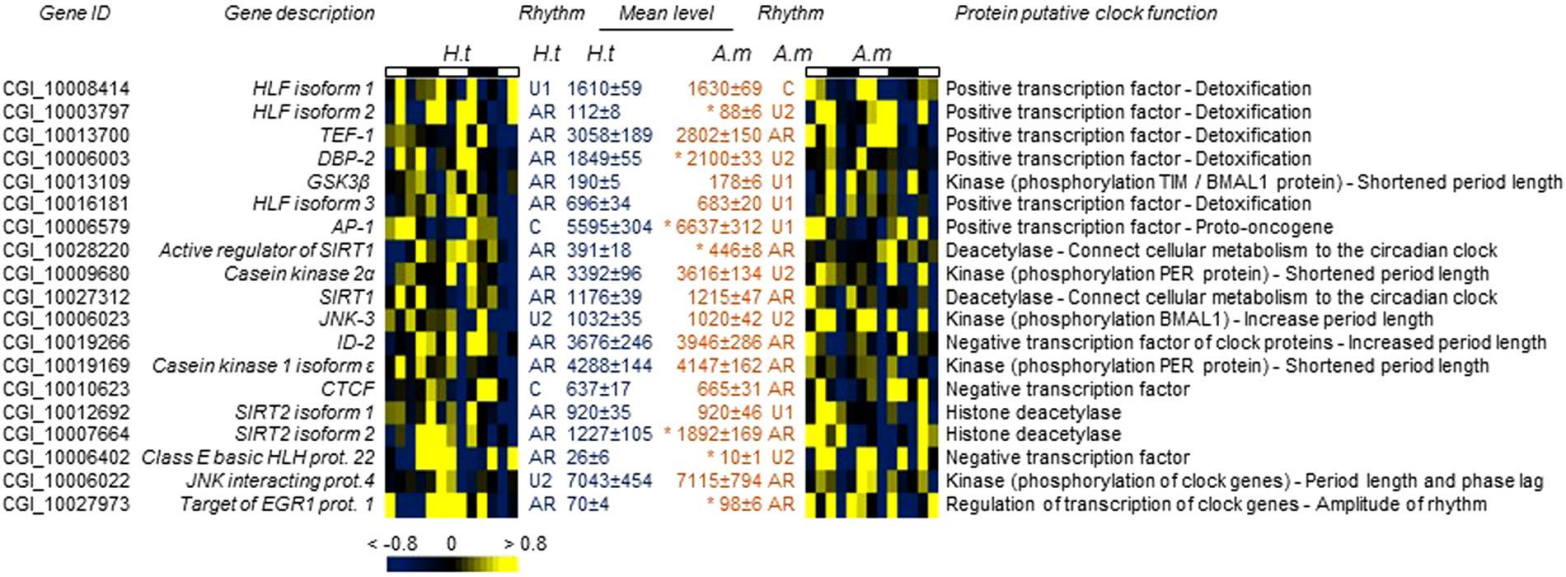
Ultradian oscillations observed in clock subsystem elements. Heat map expression of orthologous genes implicated in the clock network in mammals and insects, organized by phase of oscillation in *H.t*. “Rhythms”, results of cycling analysis (circadian, U1, U2). “Mean level”, the mean level of expression (mean ± SE). *Significant difference at *p* < 0.05.

A cycling transcriptome is the result of sophisticated regulation achieved by an integrated and complex network of core clock genes, kinases, deacetylases, and transcription factors (TFs)^2, 44, 45^. In *C. gigas* (Fig. 4), we identified 19 orthologs of vertebrate and insect genes associated with the circadian network^21, 44, 46–55^. Circadian oscillations were observed for 2 of these orthologs in *H.t*: the TFs *AP-1* and *CTCF*. Interestingly, in *H.t*, 3 transcripts were ultradian: the TF *HLF isoform 1* and the kinases *JNK-3* and *JNK interacting protein 4*. We noted a remodeling of clock elements in *A.m*, with an increase in ultradian oscillations. Indeed, in *A.m*, 9 transcripts were ultradian while only 1 transcript, *HLF isoform 1*, was circadian. In addition to remodeling the rhythmic status in *A.m*, the mean expression levels of 7 of the 19 clock network element transcripts were modified, among which 5 were increased and 2 were decreased.

### Functional implications of cycling transcripts

**Figure 5:**
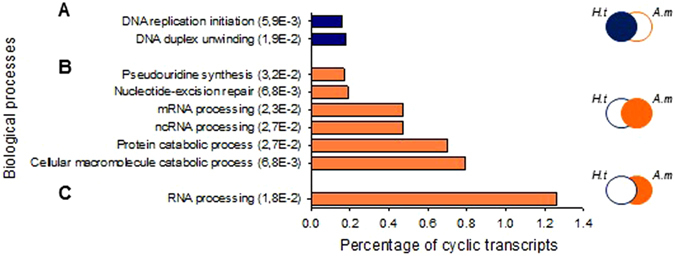
Functional implications of cycling transcripts. Enrichment analysis of biological processes of total cycling transcripts (circadian, U1, and U2) in *H.t* (**A**), in *A.m* (**B**), and uniquely in *A.m* (**C**). The ordinate axis shows the GO terms and significance (FDR) of enrichment while the abscissa axis shows the percentage of cycling for each GO term.

Overall, 5061 and 5289 transcripts oscillated (circadian and ultradian), respectively, in *H.t* and *A.m* (24.3% and 25.4% of the transcriptome, respectively (Supplementary Table S2). An enrichment analysis of the biological processes for all oscillating genes in *H.t* showed an enrichment of DNA replication initiation and DNA duplex unwinding gene ontology (GO) terms (Fig. 5A), accounting for 0.3% of total cyclic transcripts in *H.t*. The same analysis conducted with *A.m* oysters showed an enrichment accounting for 2.8% of cyclic transcripts (Fig. 5B). Pseudouridine synthesis, mRNA and ncRNA (non-coding RNA) processing, nucleotide-excision repair and catabolic processes of proteins and cellular macromolecules were enriched in the *A.m* cycling transcriptome. Finally, Fig. 5C shows that the transcripts oscillating exclusively in *A.m* enrichment in RNA processing account for 1.3%. For further analysis, Supplementary Table S3 provides the gene ID, a gene description, and their cyclic status under the other condition (*H.t* or *A.m*) for the 30 most significant circadian, U1 and U2 transcripts under *H.t* and in *A.m* conditions.

### *A. minutum* effect on gene expression level throughout the whole transcriptome

**Figure 6:**
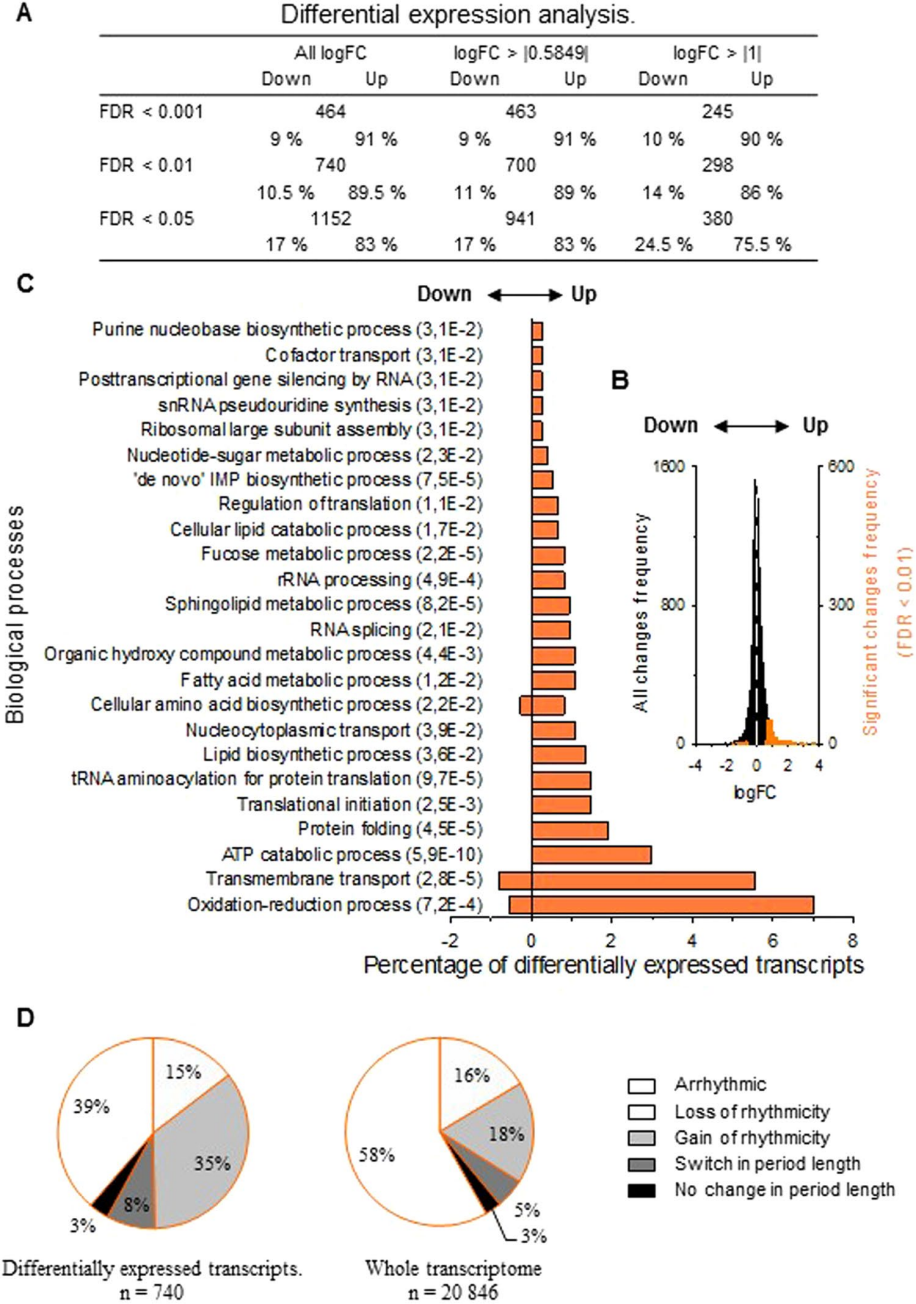
Effect of *A. minutum* on gene expression level throughout the whole transcriptome. (**A**) Number of differentially expressed (DE) transcripts in *A.m* for different significances (FDR) and fold changes (log FC). (**B**) Fold change distribution. The black bars represent equally expressed transcripts, while the orange bars represent DE transcripts. The white dotted line shows the limit between up- and downregulated transcripts. (**C**) Enrichment analysis of biological processes of DE transcripts with an FDR of <0.01. The ordinate axis shows the GO term and significance of enrichment (FDR) while the abscissa axis shows the percentage of DE transcripts enriched for each GO term. Positive and negative values indicate up- and downregulated transcripts, respectively. (**D**) The right pie chart shows the cycling transcripts’ status for the DE transcripts. The left pie chart shows the cycling transcripts’ status for the whole transcriptome in *A.m*.

Finally, we analyzed the impact of *A. minutum* exposure on the level of gene expression in the gills of *C. gigas*. Differential expression analysis was conducted using 13 time samples of each condition as replicates (summarized in Fig. 6A). At a FDR of <0.01, 740 transcripts were differentially expressed (DE); 89.5% of transcripts were upregulated and 10.5% were downregulated in *A.m* (Fig. 6A,B). Among these 740 transcripts, we conducted GO enrichment analyses to assess their functional implications (Fig. 6C). We found that in enriched biological processes, most of the transcripts were upregulated, especially those involved in the oxidative-reduction process, transmembrane transport and ATP catabolic process. Figure 6D shows a pie chart detailing the percentage of rhythmic status for the 740 DE transcripts (left pie chart) and for the whole transcriptome (right pie chart). We showed that 58% of the differentially expressed (DE) transcripts were affected by remodeling of their cyclic status compared to 39% in the whole transcriptome. This result highlighted the overrepresentation of transcripts with remodeling cycling status in differentially expressed transcripts. Specifically, transcripts with a gain of rhythmicity tend to be over-represented in DE transcripts, affecting 35% of the transcripts, compared to 18% in the whole transcriptome. Furthermore, Supplementary Fig. S3 showed the rhythmic status of the DE transcripts associated with all the significantly enriched GO terms. We noted that all the enriched GO terms were associated with remodeling of the cycling transcriptome. Finally, for further analysis, Supplementary Table S4 provides lists of the 25 most significant up- and downregulated transcripts, with their log fold change (FC) value and rhythmic status.

## Discussion

**Figure 7:**
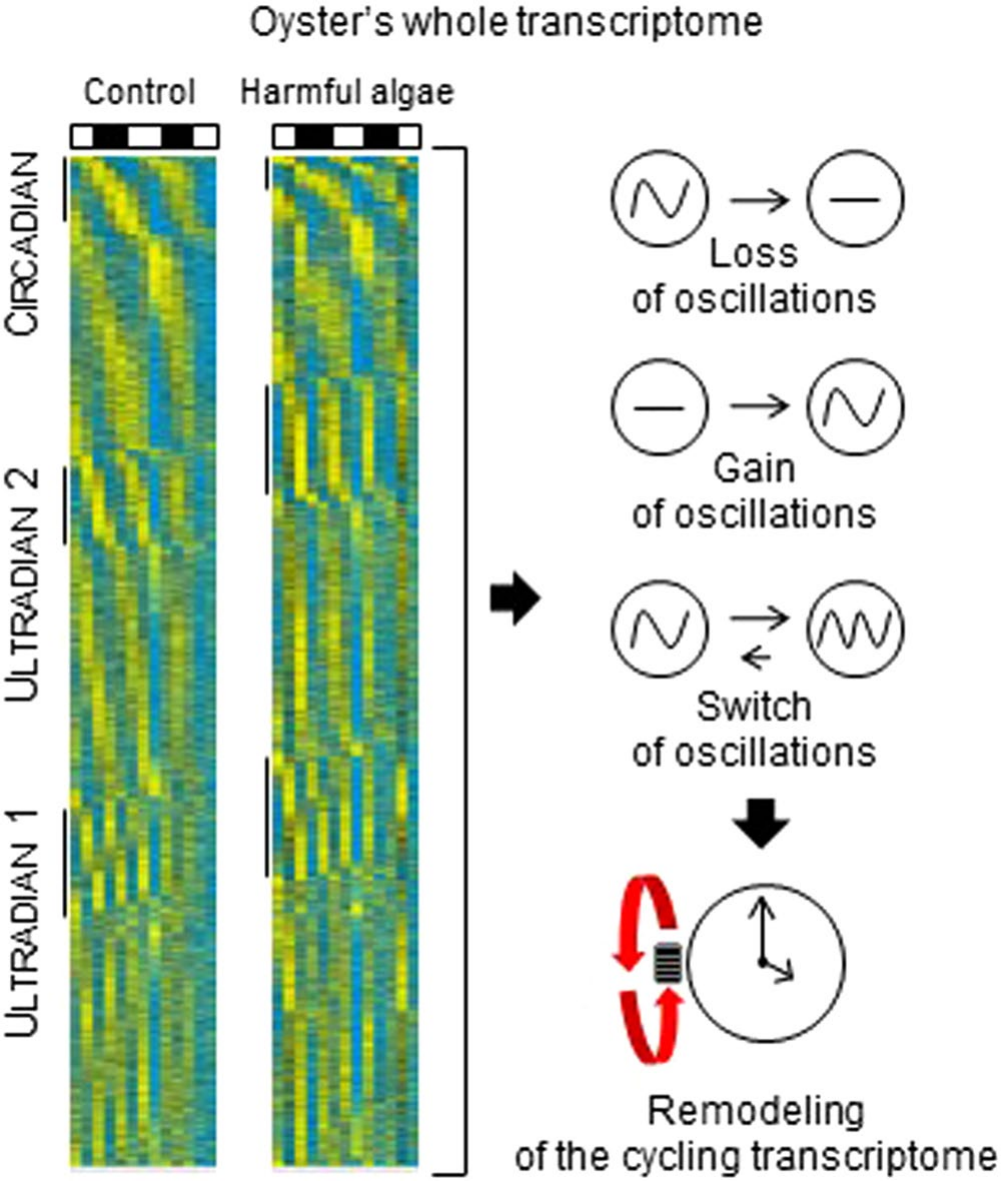
Remodeling of cycling transcriptome. Heat map expression of the whole transcriptome (20,846 transcripts), organized by significance, phase, and period in *H.t* and *A.m* respectively. Significant circadian, U1, and U2 transcripts are highlighted by a line in each condition. A summary of remodeling processes due to *A. minutum* exposure is presented on the right.

Little is known about the strategy of molecular biological rhythms in marine organisms. Our work provides significant insights for the first time, to our knowledge, into the temporal regulation of the gill transcriptome of the oyster *Crassostrea gigas*. Such gills are of importance as one of the primary tissues implicated in the mechanisms for adapting to environmental changes, such as exposure to a bloom of the PST-producer *A. minutum*. We provide evidence for the complexity and plasticity of the cycling transcriptome when oysters face a disruption in their environment. We showed that for conditions *H.t* and *A.m*, at least 42% of the different transcripts were able to exhibit rhythmicity (Supplementary Table S2). Strikingly, heat maps of the whole transcriptomes (Fig. 7) suggest wide cyclic gene expression, strongly implying a more extensive temporal organization than specifically indicated in the present work. The extensive deep sequencing effort performed in this study provide sufficient data to visualize the global oscillation of the transcriptome. Indeed, read depth sequencing is a key factor that determines the accuracy of measurements made by high-throughput sequencing, specifically for low-amplitude cyclic transcripts^19^. For example, in fruit flies^19^, characterized by a transcriptome of about 50 000 transcripts, 18 million reads per sample were estimated to be sufficient to characterize most of the circadian transcripts. According to these results, the sequencing of the 20 846 transcripts in the gills performed in this study, with a read depth of 24.5–33 million reads per sample (Supplementary Table S1), allowed to visualize possible cyclic transcripts even if the statistical analysis was not always significant. To our knowledge, the whole transcriptome organized according to the phase of the periodicity is not shown in most of published studies, likely due to a lack of sequencing effort in term of read depth analysis.

The results of the transcriptome analysis revealed circadian oscillations of 6.2% of the gill transcriptome (Fig. S1A–C). This proportion of oscillating transcripts is consistent with circadian transcript levels of 3–10% reported in specific mammalian tissues^16–22^. In the gills of the mussel *Mytilus californianus*, microarray analysis identified that ≈23% of the transcripts were circadian^3^. Circadian transcripts in our analysis were characterized by a nocturnal and bimodal pattern (Fig. 1E). Nocturnal peaks of expression for circadian genes in the gills were correlated with the expression of clock genes and the nocturnal behavior of the oysters (Fig. 2). The nocturnal pattern of behavior and core clock genes expression was in accordance to the time of year (winter) when was performed the experiment. Indeed, previous studies demonstrated the dualism of the circadian rhythm in *C. gigas*. Oyster behavior and clock gene expression profiles were nocturnal during autumn and winter^13, 40^, and diurnal during spring and summer^13, 15^. This present study, performed in winter, provided an extensive characterization of nocturnal pattern of gene expression. Although the maximum VOD occurs during the first part of the night (N1), most circadian transcripts peaked during the last part of the night (N2), when the VOD returned to its basal value (Figs 1E and 2D). These findings clearly suggested that the circadian genes in N1 and N2 are not involved in similar physiological processes. In N1, the oysters remained open most of the time, suggesting intense activity related to the surrounding environment, such as gill filtration, feeding, or respiration. By contrast, during N2, the oysters were closed most of the time, and other molecular mechanisms took over. Surprisingly, in addition to circadian transcripts, we also observed two larger groups of ultradian transcripts in *H.t*: 10.5% oscillating under a period range of 8–11 hr (U1) and 7.6% under a period range of 12–16 hr (U2). In the crustacean *Eurydice pulchra*, it has been shown that ultradian rhythms, ≈12.4-hr cycles, were driven by a dedicated circatidal pacemaker that is independent from the circadian system^10^. In *M. californianus* exposed to simulated daily and tidal entrainments, ≈2% of transcripts oscillated at a periodicity of 10–14 hr^3^. However, no endogenous circatidal rhythm has been demonstrated in *C. gigas* ^14^, and oysters in this experiment were not exposed to ultradian zeitgeber, such as tidal cues. Several studies have identified clock transcripts with an ultradian period of 12 hr in the mouse liver^18, 49, 56–58^. A system involving a layered circadian transcriptional cascade has been proposed, which could lead to the generation of ultradian genes by an interaction with TFs, which is in turn associated with the induction of clock genes and CCGs^45^. In this study, we identified oyster orthologs of the mammalian and insect circadian subsystems, such as TF *HLF isoform 1*, kinase *JNK 3*, and *JNK interacting protein 4*, which oscillated with an ultradian periodicity, suggesting that the oyster circadian network was involved in ultradian rhythmicity (Fig. 4, *H.t*). The unusual period of ultradian transcripts (U1 and U2) may be explained by the 9 L: 15D regime, mimicking the winter photoperiod observed in the field at the time of the experiment. Indeed, both periods of oscillation, U1 and U2, were close to the unequal durations of light and night (9 L: 15D) (Fig. 3A). This regime differed from the classical 12 L: 12D cycle used in most chronobiological studies.

HABs are currently increasing due to concomitant factors such as global warming, world shipping, and agriculture, and exert a significant effect in terms of ecological and physiological consequences on marine organisms such as filter-feeding bivalves^33^. In oysters, much damage has been shown in terms of genotoxicity, lipid metabolism impairment, immunotoxicity, increase of mucus in gills, myopathies, and modifications of filtration and valve behavior^14, 34–39, 59, 60^. Here, we discovered an important remodeling of the cycling transcriptome. Compared to *H.t*, *A. minutum* exposure led to (1) a loss of 68% of previous cyclic transcripts; (2) a gain of 69% of *de novo* cyclic transcripts; and (3) a switch of periodicity in 21% of cyclic transcripts, as shown in Supplementary Table S2. At the circadian level, exposure to *A. minutum* led to a ≈50% decrease in the number of cyclic transcripts, with a modification in phase lag and a loss of cyclic transcripts with low amplitudes (Fig. 1, Supplementary Table S2). The modification of phase lag expression was associated with a loss in nocturnal and bimodal pattern, leading to the absence of a clear pattern of daily expression (Fig. 1E–G). Similar trends were observed in the expression of clock genes and valve opening behavior (Fig. 2). As explain earlier, diurnal and nocturnal behavioral patterns alternate in *C. gigas* throughout the year in a predictable way13. This dualism could be an adaptive response to variations in energy needs during the year^13^. Consequently, the disruption of the nocturnal circadian pattern observed in this study could lead to a loss of fitness of the oyster within its biotope. Note, in *A.m*, we observed an increase in *Target of EGR1* expression (Fig. 4). As *EGR1* is involved in the amplitude of circadian transcripts in the mouse^51^, *Target of EGR1* could be implicated in the increase of circadian transcripts with high amplitudes in *A.m* (Fig. 1I). Remodeling of circadian gene profiles (loss and gain of oscillation, as well as modification of phase lag expression) has been previously demonstrated in the mouse. Evidence for a modulatory role of the transcriptional factor *KLF15* in the diurnal pattern of the circadian genes in the mouse heart has been shown^61^. Indeed, in the heart of KLF15 knockout mice, authors observed 805 circadian transcripts compared to 1335 in control heart, with only 332 common circadian transcripts. Authors demonstrated that KLF15 was involved in the generation of transcripts oscillations and biphasic patterns as well as in the inhibition of oscillations of others transcripts and the quiescent expression of other genes associated to the circadian subsystem^61^. Similar weak number of commonly detected circadian transcripts was observed in liver of mice fed with high fat diet compared to normal diet^17^. This reprogramming involved both an impairment of CLOCK-BMAL chromatin recruitment and a cyclic activation of surrogate pathway through the transcriptional regulator PPAR γ^17^.

At the ultradian level, we observed a 24% increase in ultradian transcripts in *A.m* compared to *H.t* (Supplementary Table S2). An analysis of oyster orthologs of TFs and kinases involved in the circadian network of mammals and insects also showed an increase in ultradian oscillations in *A.m* (Fig. 4). Interestingly, the circadian TFs *HLF* and *DBP2* are known to control the expression of enzymes and regulators involved in detoxification and drug metabolism in mice^62, 63^. In our study, *HLF isoform 2*, *HLF isoform 3*, and *DBP2*, which were arrhythmic in *H.t*, exhibited an ultradian oscillation in *A.m*. Thus, the increase of ultradian transcripts in *A.m* could be linked to the implementation of detoxification mechanisms in response to PSTs bioaccumulation, through the TF *HLFs* and *DBP2*. The TF ortholog of *AP-1* was observed to switch from circadian to U1 oscillation in *A.m*. This result suggested an acceleration of cell proliferation, apoptosis, and abnormal cell cycles, *i.e*. cellular processes in which *AP-1* is a regulator^64^. The kinase *GSK-3β* and casein kinase 2α, which are also arrhythmic in *H.t*, gained ultradian rhythmicity. Both kinases are known to act on TIM or BMAL1 phosphorylation and could induce a shortened period of cyclic processes, offering interesting leads to explain the decrease of U2 period length observed in *A.m* ^47, 65, 66^.

In addition to the remodeling of the cycling transcriptome, *A. minutum* exposure led to a change in the expression level of 740 transcripts (FDR of <0.01), with 89.5% exhibiting overexpression (Fig. 6), suggesting physiological reactions against this harmful alga. Fig. 6D revealed that 58% of differentially expressed transcripts appeared to be affected by cyclic remodeling. Moreover, all enriched biological processes (GO terms) were impacted by a remodeling of the cyclic transcriptome (Supplementary Fig. S3), suggesting a pleiotropic impact of *A. minutum*.

Mechanisms associated to the remodeling of cyclic transcriptome of *C. gigas* during *A. minutum* exposure remained unknown. *Alexandrium minutum* is known to produce paralytic shellfish toxins (such as STX) but also bioactive extracellular compounds that can exhibit toxic activities by direct contact with gills^67^. An effect of *A. minutum* directly on core clock genes or on the clock subsystem genes, through genotoxic impacts could be suggested, as previously observed^14^. Moreover, previous studies on chronotoxic effects in mammals and mollusk gastropods such as *Aplysia* have shown that the potential action of clock neurons was blocked by TTX, a homologue of STX toxin. The master clock kept time, but did not send the circadian message to other regions^68, 69^. Another study reported that TTX damped and desynchronized the suprachiasmatic nucleus cellular rhythms in *Per1* expression^70^.

To conclude, this original study of the temporal transcriptome of *C. gigas* revealed highly cyclic gene expression and highlighted the complexity of clock mechanisms, with an intriguing predominance of ultradian genes in a circadian context. Moreover, the results showed pleiotropic impacts of *A. minutum* exposure on the oyster gill transcriptome. The transcriptional remodeling associated with exposure to harmful algae revealed plasticity in the temporal organization of gene expression. To date, the consequences of this remodeling plasticity remain unknown. This response could be an adaptive reaction to the toxicity of the harmful algae. It may also reveal a consequent chronotoxicity effect leading to a disruption in the temporal structure and an uncoupling of the transcriptional rhythmicity associated with the physiological needs. In any case, this effect led to a loss of fitness of the oyster within its environment.

### Experimental procedures

#### Animals and experimental treatment

Diploid Pacific *C. gigas* oysters (1.5 years old, n = 156) from the Bay of Arcachon (France) (72.8 ± 0.5 mm shell length; 40.6 ± 0.4 mm shell width; 38.5 ± 0.8 g total fresh weight; mean ± SE) were divided among 6 tanks isolated from external vibration using an anti-vibrating bench and continuously supplied with homogenized seawater of constant composition (T °C = 15.0 ± 0.2 °C; pH = 8.0 ± 0.1; salinity = 35.8 ± 0.01‰). Experiments were conducted in December to avoid gametogenesis. Oysters were acclimated for 7 days to 9 hr light-15 hr dark conditions mimicking the natural light regime in winter time fed with the non-harmful alga *Heterocapsa triquetra* of pre-exposure (continuously supplied with algae at a flow rate of 100 mL.min^−1^ at a concentration of 1000 cells.mL^−1^; 208.3 ± 44.5 cells.mL^−1^ measured in *H.t* tanks; 208.7 ± 38.1 cells.mL^−1^ measured in the *A.m* tanks, mean ± SE). Then, the *H.t* treatment group (3 replicate tanks) remained exposed to *H. triquetra* whereas the *A.m* treatment group (3 replicate tanks) was exposed to the harmful alga *Alexandrium minutum* for 6 days (continuously supplied with algae at a flow rate of 100 mL.min^−1^ at a concentration of 1000 cells.mL^−1^; 159.1 ± 15.7 cells.mL^−1^ measured in *H.t* tanks; 430.9 ± 160.8 cells.mL^−1^ measured in the *A.m* tanks, mean ± SE). During the two last days of exposure, gill tissue (n = 6 oysters per condition) was collected every 4 hr for 52 hr, under natural light during light phases and under dim red light during dark phases, for a total of 13 sampling times (day 1: Zeitgeber time ZT 4.5, ZT 8.5, ZT 12.5, ZT 16.5, ZT 20.5; day 2: ZT 0.5, ZT 4.5, ZT 8.5, ZT 12.5, ZT 16.5, ZT 20.5; day 3: ZT 0.5; ZT 4.5). Zeitgeber time 0 (ZT0) corresponds to the light switch-on.

### Algae

The paralytic shellfish toxin-producing dinoflagellate *A. minutum* (strain AM89BM, Halim) and the non-toxic dinoflagellate *H. triquetra* (strain HT99PZ –Ehrenberg, 1840) were grown in 10 L and 80 L phytoreactors using f/2 medium^71^. During the exponential growth phase, *A. minutum* produced 1.3 ± 0.1 pg saxitoxin per cell^72^. *Heterocapsa triquetra* was chosen as control for several reasons. As *A. minutum*, *H. triquetra* belonged to the dinoflagellates and both species exhibited similar characteristics of shape and size with 19–28 μm and 23–29 μm cell sizes for *H. triquetra* and *A. minutum*, respectively. Algae concentrations were assessed by a Beckman Coulter Z2 (Beckman Coulter Inc., USA).

### Paralytic Shellfish Toxins (PST)

Measurement of PST content in gill tissues of animals used for RNA-seq analysis was done during the 52 hr of sampling (n = 72). PST (saxitoxin and its derivatives) were assessed by ELISA (Abraxis, Novakits, France) according to manufacturer’s instructions. Briefly, individual samples were crushed on ice, centrifuged, and diluted. Standard solutions and samples were added in duplicate (50 µl) into wells of an ELISA microtiter plate coated with a secondary sheep anti-rabbit antibody. Then, the saxitoxin horseradish peroxidase (saxitoxin-HRP) conjugate solution and the primary antibody solution (rabbit anti-saxitoxin) were added successively. Plates were incubated at room temperature and washed four times using washing solution. Substrate solution containing tetramethyl-benzidine was added to each well and plates were incubated. Finally, reactions were stopped by a solution containing sulfuric acid and absorbance was read at 450 nm. Color intensity was inversely proportional to the concentration of PSTs in the samples.

### RNA sequencing (RNAseq)

Total RNA from gills was extracted from samples using TRI® Reagent (Invitrogen). After purity (Nanodrop) and quality (Agilent 210 BioAnalyzer, RIN value > 8.5) checks, RNA samples were normalized and pooled (n = 6) in equal quantities of total RNA, according to time and condition. Messenger RNA purifications were performed to generate 26 libraries from 4 µg of total RNA using the Illumina TruSeq Strand mRNA Sample Prep kit according to the manufacturer’s protocol. Sequencing of 100 bp paired-end fragments was performed on four pooled libraries, each one on two lanes, on the same flowcell, using an Illumina Hiseq 2500 at the genomic platform Génopole Toulouse/Midi-Pyrénées (INRA Auzeville, France).

### RNAseq data analysis

Adapter and base quality trimming (Phred score < 20) was performed with Trim Galore (trim_galore_v0.3.7). The read depth was fixed at 47.0 million reads/sample (down-sampling normalization, Supplementary Table S1) for cycling transcriptome analysis, according to^19^. Relative log expression normalization (RLE)^73^ was applied instead of down-sampling for differential expression analysis (edgeR version 3.8.6^74^). Sequenced reads were aligned by STAR (STAR_2.4.2a^75^) to both the genome and mitochondrial genome of *Crassostrea gigas* ^76^, Ensemble 1.30; and NCBI reference NC_001276) and assigned by FeatureCount (1.4.5 version^77^) to annotations of the genome in public databases. Expression levels of genes were presented as cpm (counts per million). Filtering of transcripts with 0 cpm in the whole set of libraries was applied. Indeed, considering the homogenized read depth of samples (Supplementary Table S2), 0 cpm value for a transcript that have at least 1 cpm in at least one library was considered as a low expression and not a missing data. Finally, a total of 20,846 and 20,796 transcripts were obtained for analysis of cycling transcripts and differentially expressed transcripts, respectively.

### Analysis of transcripts

Sequencing outputs and bioinformatics treatments were carefully analyzed and checked several times to ensure the reliability of data supporting the discussion. Cycling transcripts were detected using ARSER^41^ with MetaCycle (version 1.1.0^78^ on R^79^ (32-bit, version 3.2.2)). ARSER algorithm was developed and applied to high-throughput time-series analysis and particularly adapted to experimental designs with a timeframe of 4 h over 52 hr^41, 78, 80^. Post-hocs were corrected for multiple comparisons to remove false discoveries^42, 43^. For all cycling detection, a false discovery rate (FDR) of <0.05 was considered significant. Note that in 24 hr light/dark entrainment, the external circadian cues may directly generate 24 hr rhythms, in an addition of CCGs group. Amplitudes of oscillating transcripts were calculated as ((high value - low value)/low value)^57^. All these genes with a period length between 20 hr and 28 hr comprise circadian transcripts^45^. Differentially expressed transcripts were analyzed with edgeR (edgeR version 3.8.6), considering the 13 sample times (day 1 ZT 0.5 to day 3 ZT 0.5) of each algal treatment as replicates; the results were corrected for multiple tests using the FDR^81^.

### Gene ontology analysis

Gene descriptions were obtained using the *C. gigas* reference genome^76^ on Biomart, Ensembl Metazoa (http://metazoa.ensembl.org/biomart/martview/). Enrichment analyses were performed using the Fisher test on Blast2GO software (version 2.8), selecting only specific biological processes.

### cDNA synthesis and real-time quantitative PCR (RT-qPCR)

The same samples used for RNA sequencing were analyzed by performing RT-qPCR at 7 sampling times (day 2: ZT 0.5, ZT 4.5, ZT 8.5, ZT 12.5, ZT 16.5, ZT 20.5 and day 3: ZT 0.5). Total RNA from individual samples of gills was extracted as previously described and reverse transcribed using oligo dT17 and Moloney murine leukemia virus reverse transcriptase (M-MLV; Promega). RT-qPCR reactions were performed in replicate (n = 6) on individual samples using Brillant III Ultra-Fast SYBR® Green QPCR Master Mix kit (Agilent)^15^. Primer sets of circadian clock genes were designed^15^ and are listed in Supplementary Table S5. EF1 (Elongation factor 1) was selected as the housekeeping gene using geNorm, BestKeeper and Normfinder (Wang *et al*. 2012) and relative expression levels were calculated using the comparative Ct method (2^−ΔΔCt^ method^82^). Validation of the RNAseq analysis was done by RT-qPCR assessment of clock genes. Normalization of gene expression was done for both analyses as follow: (X_i_ - min)/(max - min), where X_i_ was the gene expression value, min and max were the minimum and maximum values of the gene expression in a given condition and for a specific clock gene (Supplementary Fig. S2).

### Behavioral monitoring

The valve activity of *C. gigas* (n = 32) was studied using two HFNI (high-frequency non-invasive) valvometers^40, 83^. Lightweight electromagnets (0.1 g) were glued on each valve of each animal. These electrodes were connected to the valvometer by flexible wires which allowed the oysters to move their valves without constraint. The sampling frequency for each individual was 0.2 Hz. Data were processed using LabView 8.0 software (National Instruments). For the VOD, the mean hourly valve opening duration was expressed as the percentage of time that oysters spent with their valves open: 100% indicates that the valves were open for the entire hour and 0% indicates that the valves were closed for the hour. The 48-hr mean hourly opening durations for 16 oysters per condition was used in the analyses.

### Statistical analysis for RT-qPCR and behavioral analysis

Results are expressed as the means ± the SE. Treatment differences were determined using analysis of variance (ANOVA) after checking assumptions (normality and homoscedasticity of the error term). When assumptions were not met, the non-parametric Kruskall–Wallis test was performed. If the null hypothesis was rejected, the Student –Newman-Keuls method was applied to determine significant differences between conditions. For all statistical results, a probability of p < 0.05 was considered significant. Statistical analyses were performed using Sigma Stat software (Version 13.0, SYSTAT, Chicago, USA).

### Data access

RNAseq data are available in the ArrayExpress database (www.ebi.ac.uk/arrayexpress) under accession number E-MTAB-5345.

## Electronic supplementary material

Supplementary Information

Supplementary Table S3
