# Supplementary material for: Remodeling of the cycling transcriptome of the oyster Crassostrea gigas by the harmful algae Alexandrium minutum
Source: Sci Rep. 2017 Jun 14;7:3480. doi: 10.1038/s41598-017-03797-4 (PMC5471176; doi:10.1038/s41598-017-03797-4)
Supplement: Supplementary file 1 — Supplementary Information [file 41598_2017_3797_MOESM1_ESM.pdf]

Supplementary information

Remodeling of the cycling transcriptome of the oyster *Crassostrea gigas* by the harmful algae *Alexandrium minutum*.

Laura Payton[1,2], Mickael Perrigault[1,2], Claire Hoede[3], Jean-Charles Massabuau[1,2], Mohamedou Sow[1], Arnaud Huvet[4], Floriane Boullot[4,5], Caroline Fabioux[4,5], Hélène Hegaret[4,5], Damien Tran[1,2*]

[1]University of Bordeaux, EPOC, UMR 5805, F-33120 Arcachon, France

[2]CNRS, EPOC, UMR 5805, F-33120 Arcachon, France

[3]Plate-forme bio-informatique Genotoul, MIAT, Université de Toulouse, INRA, F-31326 Castanet-Tolosan, France.

[4]Ifremer, Laboratoire des Sciences de l'Environnement Marin (LEMAR), UMR 6539 UBO/CNRS/IRD/IFREMER), CS 10070, F-29280 Plouzané, France

[5]Laboratoire des Sciences de l'Environnement Marin (LEMAR), Institut Universitaire Européen de la Mer, Université de Bretagne Occidentale, UMR 6539 CNRS/UBO/IRD/IFREMER, F-29280 Plouzané, France

* to whom correspondence should be addressed. UMR 5805 EPOC, Place du Dr Peyneau, 33120, Arcachon, France. E.mail: d.tran@epoc.u-bordeaux1.fr. Tel : +33 562239237

Supplementary Figures

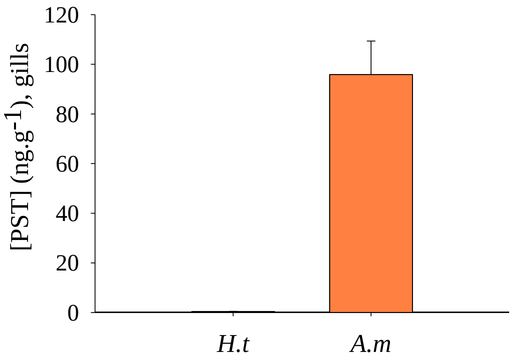

Supplementary Figure S1. PST toxins bioaccumulation in gills.

Quantification of PST bioaccumulation (ng.g^{-1} eqSTX, mean ± SE, n = 72) in gills of *H.t* and *A.m C. gigas*.

A

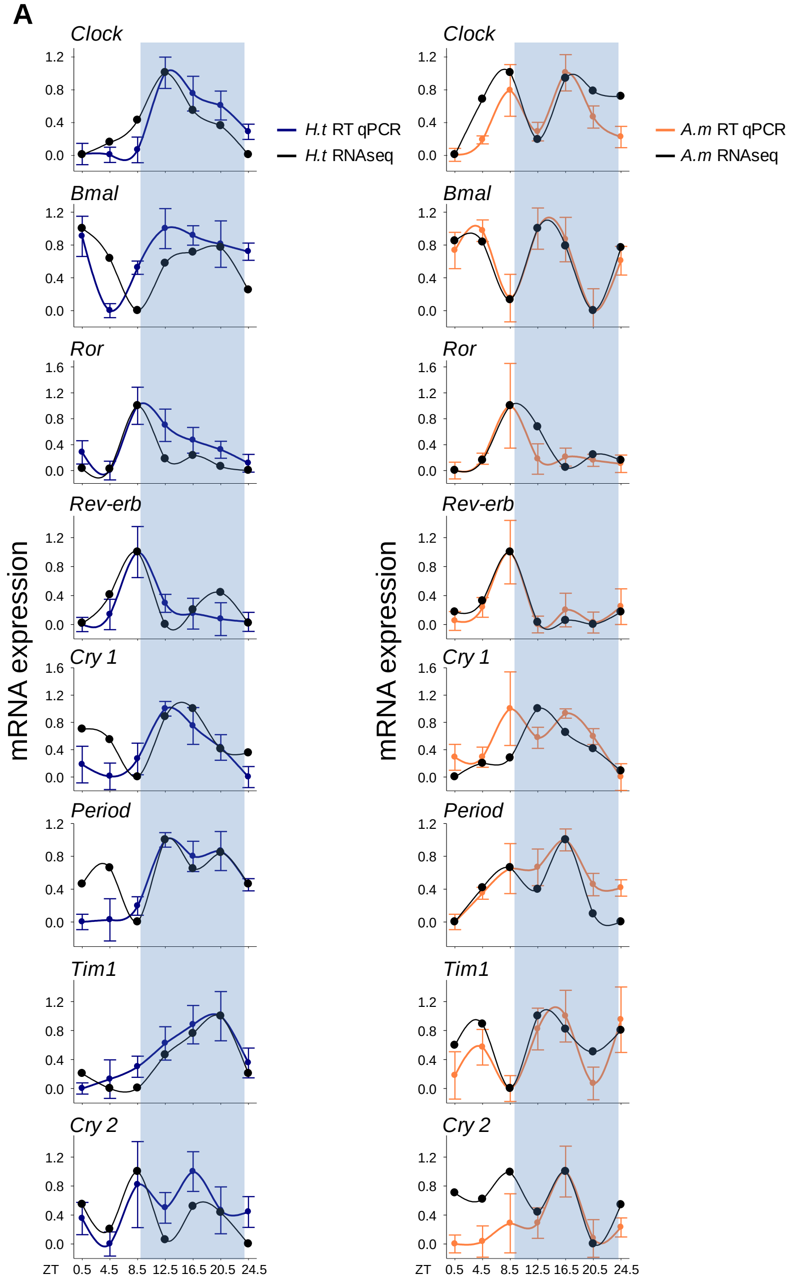

B

	Clock	*Bmal*	*Ror*	*Rev-erb*	*Cry 1*	*Period*	*Tim1*	*Cry 2*	Total *H.t*	Total *A.m*
R	0.749	0.655	0.776	0.886	0.468	0.678	0.803	0.352	0.673	0.720
p-value	0.0020	0.0110	0.0011	> 0.0001	0.0913	0.0078	0.0005	0.2170	> 0.0001	> 0.0001

Supplementary Figure S2. Circadian core clock gene expression quantified by both real-time qPCR and RNAseq. (**A**) Relative expression measured with real-time qPCR (mean ± SE) were compared with RNAseq approach in *H.t* (on the left) and *A.m* (on the right) (normalized expression). Gray areas correspond to nighttime. (**B**) Pearson correlation results between both analysis (normalized data) on each circadian clock genes (*H.t* and *A.m* confounded) and on the all gene expression in *H.t* and *A.m* condition respectively.

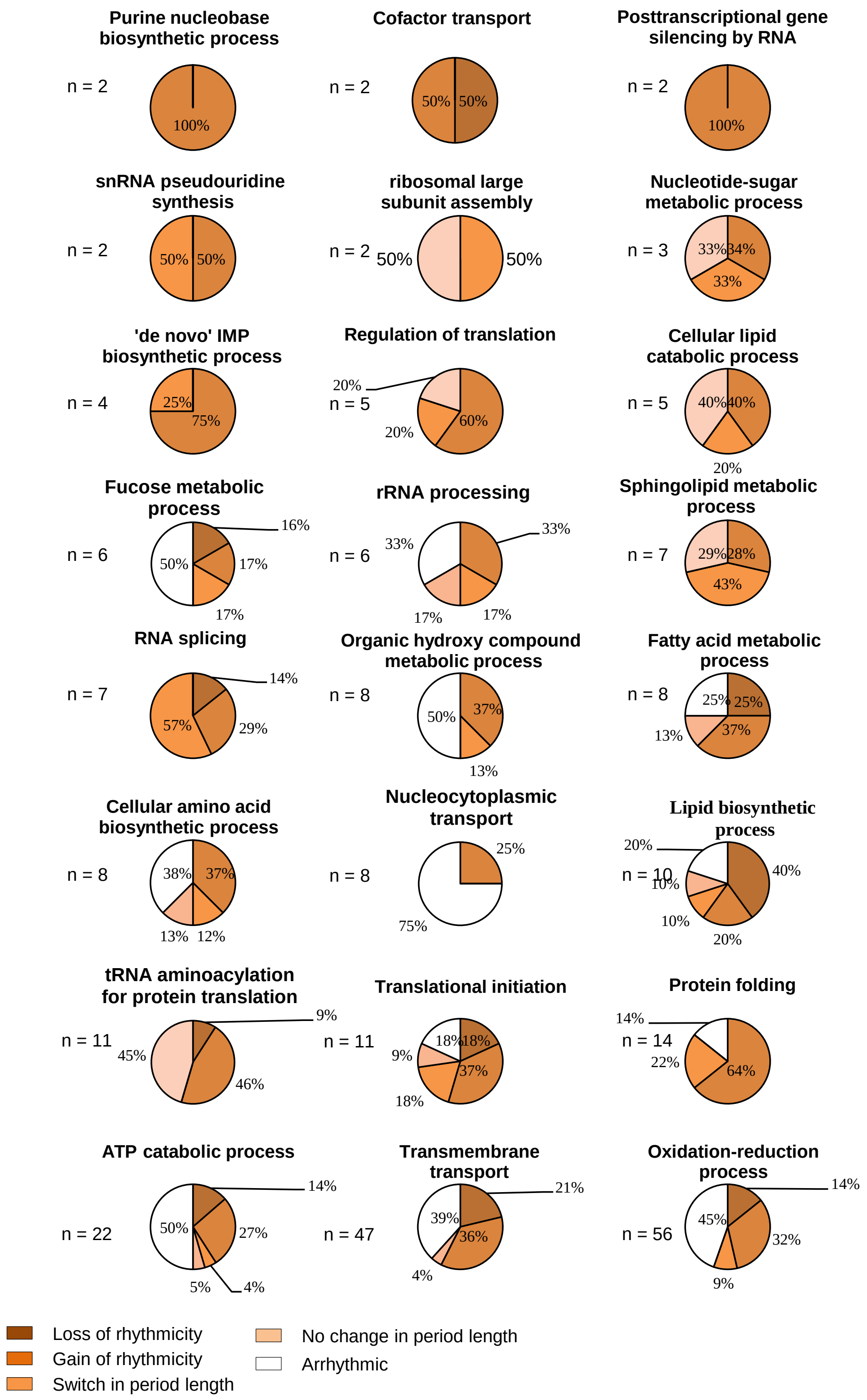

Supplementary Figure S3. Details of cycling status for enriched biological processes of DE analysis.
“n”: the number of transcripts affected by GO terms.

Supplementary Tables

Supplementary Table S1. Details of RNAseq analyzes.

Time	Sample	Total reads, millions	Aligned reads, millions (STAR) (percentage of total reads)	Assigned reads, millions (FeatureCount) (percentage of aligned reads)
Analysis of cycling transcripts, Down Sampling normalization.				
day 1 ZT 4.5	1 *H.t*	47	45.2 (96.2 %)	27.0 (59.8 %)
day 1 ZT 8.5	2 *H.t*	47	45.8 (97.4 %)	27.8 (60.8 %)
day 1 ZT 12.5	3 *H.t*	47	45.5 (96.8 %)	27.6 (60.6 %)
day 1 ZT 16.5	4 *H.t*	47	45.9 (97.7 %)	27.8 (60.4 %)
day 1 ZT 20.5	5 *H.t*	47	45.9 (97.7 %)	27.0 (58.9 %)
day 2 ZT 0.5	6 *H.t*	47	45.6 (97.0 %)	27.2 (59.6 %)
day 2 ZT 4.5	7 *H.t*	47	45.9 (97.7 %)	27.4 (59.9 %)
day 2 ZT 8.5	8 *H.t*	47	51.9 (100.0 %)	33.1 (63.7 %)
day 2 ZT 12.5	9 *H.t*	47	45.7 (97.2 %)	27.6 (60.4 %)
day 2 ZT 16.5	10 *H.t*	47	45.9 (97.7 %)	27.7 (60.3 %)
day 2 ZT 20.5	11 *H.t*	47	46.1 (98.1 %)	27.2 (58.9 %)
day 3 ZT 0.5	12 *H.t*	47	44.7 (95.1 %)	24.5 (54.8 %)
day 3 ZT 4.5	13 *H.t*	47	45.5 (96.8 %)	25.0 (54.9 %)
day 1 ZT 4.5	1 *A.m*	47	46.3 (98.5 %)	28.2 (60.9 %)
day 1 ZT 8.5	2 *A.m*	47	46.3 (98.5 %)	28.4 (61.2 %)
day 1 ZT 12.5	3 *A.m*	47	46.4 (98.7 %)	29.3 (63.0 %)
day 1 ZT 16.5	4 *A.m*	47	42.5 (90.4 %)	25.4 (59.7 %)
day 1 ZT 20.5	5 *A.m*	47	48.5 (100.0 %)	29.3 (60.5 %)
day 2 ZT 0.5	6 *A.m*	47	46.5 (98.9 %)	28.6 (61.5 %)
day 2 ZT 4.5	7 *A.m*	47	45.9 (97.6 %)	27.9 (60.8 %)
day 2 ZT 8.5	8 *A.m*	47	53.4 (100.0 %)	33.9 (63.4 %)
day 2 ZT 12.5	9 *A.m*	47	46.6 (99.1 %)	29.0 (62.3 %)
day 2 ZT 16.5	10 *A.m*	47	46.1 (98.1 %)	26.2 (56.9 %)
day 2 ZT 20.5	11 *A.m*	47	46.3 (98.5 %)	26.5 (57.2 %)
day 3 ZT 0.5	12 *A.m*	47	46.3 (98.5 %)	26.7 (57.8 %)
day 3 ZT 4.5	13 *A.m*	47	46.1 (98.1 %)	26.2 (56.7 %)
Analysis of differential expressed transcripts, Relative Log Expression (RLE) normalization.				
day 1 ZT 4.5	1 *H.t*	62.6	60.4 (96.5 %)	36.2 (59.8 %)
day 1 ZT 8.5	2 *H.t*	67.1	65.5 (97.6 %)	39.8 (60.8 %)
day 1 ZT 12.5	3 *H.t*	65	63.1 (97.1 %)	38.3 (60.6 %)
day 1 ZT 16.5	4 *H.t*	68.5	67.1 (98.0 %)	40.6 (60.5 %)
day 1 ZT 20.5	5 *H.t*	59.2	57.9 (97.8 %)	34.2 (59.0 %)
day 2 ZT 0.5	6 *H.t*	59	57.5 (97.6 %)	34.3 (59.7 %)
day 2 ZT 4.5	7 *H.t*	69.4	68.0 (98.0 %)	40.8 (60.0 %)
day 2 ZT 8.5	8 *H.t*	62.8	69.6 (100.0 %)	44.4 (63.8 %)
day 2 ZT 12.5	9 *H.t*	62.3	60.8 (97.6 %)	36.8 (60.5 %)

day 2 ZT 16.5	10 *H.t*	47	45.9 (97.7 %)	27.7 (60.3 %)
day 2 ZT 20.5	11 *H.t*	73.4	72.2 (98.4 %)	40.3 (55.9 %)
day 3 ZT 0.5	12 *H.t*	54.6	52.0 (95.2 %)	28.1 (53.9 %)
day 3 ZT 4.5	13 *H.t*	51.3	49.7 (97.0 %)	27.1 (54.4 %)
day 1 ZT 4.5	1 *A.m*	87.6	86.4 (98.6 %)	52.6 (60.9 %)
day 1 ZT 8.5	2 *A.m*	61.2	60.5 (98.9 %)	37.1 (61.2 %)
day 1 ZT 12.5	3 *A.m*	60.5	59.9 (99.0 %)	37.8 (63.1 %)
day 1 ZT 16.5	4 *A.m*	51.3	46.4 (90.4 %)	27.7 (59.7 %)
day 1 ZT 20.5	5 *A.m*	70.8	73.2 (100.0 %)	44.3 (60.5 %)
day 2 ZT 0.5	6 *A.m*	71	70.3 (99.0 %)	43.2 (61.5 %)
day 2 ZT 4.5	7 *A.m*	62.4	45.8 (73.4 %)	27.3 (59.6 %)
day 2 ZT 8.5	8 *A.m*	65.9	75.1 (100.0 %)	47.6 (63.4 %)
day 2 ZT 12.5	9 *A.m*	63.4	63.1 (99.5 %)	39.3 (62.3 %)
day 2 ZT 16.5	10 *A.m*	60.6	59.5 (98.2 %)	33.0 (55.9 %)
day 2 ZT 20.5	11 *A.m*	66.4	65.6 (98.8 %)	36.1 (55.0 %)
day 3 ZT 0.5	12 *A.m*	61.8	61.0 (98.7 %)	34.2 (56.0 %)
day 3 ZT 4.5	13 *A.m*	54.7	53.8 (98.3 %)	30.1 (55.9 %)

Supplementary Table S2. Number of cycling transcripts (ARSER, FDR < 0.05) in *H.t* and *A.m* condition, details of *A.m* exposure's effect on cycling transcripts (number).

Period range	Cycling transcripts in *H.t* (% among total transcripts)	Cycling transcripts in *A.m* (% among total transcripts)	Details of *A.m* exposure's effect on cycling transcripts			
			No change in period length	Switch in period length range	Loss of rhythmicity	Gain of rhythmicity
Circadian (20-28 hr)	1300 (6.2 %)	630 (3.0 %)	53	358	889	428
Ultradian 1 (8-11 hr)	2185 (10.5 %)	2452 (11.8 %)	294	400	1491	1737
Ultradian 2 (12-16 hr)	1576 (7.6 %)	2207 (10.6 %)	228	300	1048	1484
All period range	5061 (24.3 %)	5289 (25.4 %)	575	1058	3428	3649

Number of total transcripts: 20846

Supplementary Table S4. The 25 transcripts most significantly up and down regulated with *Am* condition. "FDR": significance and fold change of differential expression analysis; "*H.t*": cycling status of transcripts in *H.t*; "*A.m*": cycling status of transcripts in *A.m* (AR, arrhythmic; C, circadian; U1, ultradian 1; U2, ultradian 2).

Gene ID	Gene description	FDR	logFC	*H.t*	*A.m*
	Up-regulated transcripts				
CGI_10017999	Multidrug resistance protein 1	5.4E-46	3.36	AR	AR
CGI_10017108	Tyramine beta-hydroxylase	7.7E-38	3.67	AR	AR
CGI_10017107	Temptin	2.5E-37	3.40	AR	AR
CGI_10001849	Dopamine beta-hydroxylase	9.1E-35	3.55	AR	AR
CGI_10027142	Nose resistant to fluoxetine protein 6	3.7E-34	3.48	AR	U2
CGI_10002724	Arylacetamide deacetylase	5.4E-30	2.29	U2	U1
CGI_10020743	AOX, Alternative oxidase, mitochondrial	2.5E-29	3.53	AR	AR
CGI_10009284	von Willebrand factor D and EGF domain-containing protein	8.2E-29	2.99	AR	AR
CGI_10021001	Inositol-3-phosphate synthase 1-B	3.3E-28	2.54	AR	U2
CGI_10022749	Glutathione-requiring prostaglandin D synthase	2.3E-27	2.52	U1	AR
CGI_10026302	CCAAT/enhancer-binding protein delta	4.1E-27	3.76	AR	U2
CGI_10005728	Cholinesterase	2.5E-26	3.23	AR	AR
CGI_10022331	Steroid 17-alpha-hydroxylase/17,20 lyase	2.7E-25	1.88	U2	AR
CGI_10010435	Vacuolar protein sorting-associated protein 4B	5.5E-25	1.98	AR	AR
CGI_10002314	Short chain dehydrogenase/reductase family 9C member 7	3.8E-23	4.74	AR	AR
CGI_10005726	Neuroligin-4, X-linked	2.1E-21	2.30	U1	AR
CGI_10026674	DBH-like monooxygenase protein 1	3.0E-21	3.85	AR	C
CGI_10018845	Periostin	1.1E-20	2.67	U1	U2
CGI_10027533	General transcription factor 3C polypeptide 1	2.0E-20	2.17	AR	AR
CGI_10002509	Steroid 17-alpha-hydroxylase/17,20 lyase	1.3E-19	2.11	AR	AR
CGI_10008887	Solute carrier family 15 member 4	1.1E-18	1.62	AR	AR
CGI_10016297	WW domain-containing oxidoreductase	2.8E-18	1.58	AR	AR
CGI_10016303	Cytochrome P450 3A11	5.1E-18	2.07	AR	AR
CGI_10002510	Cytochrome P450 1A1	5.8E-18	2.07	AR	AR
CGI_10021884	GRAM domain-containing protein 4	1.2E-16	1.52	AR	U2
Down-regulated transcripts					
CGI_10024446	Aquaporin-2	3.4E-15	-2.08	AR	U2
CGI_10021193	Caveolin	1.3E-06	-1.17	AR	AR
CGI_10025965	Ankyrin repeat and SAM domain-containing protein 1A	2.4E-06	-6.10	U2	AR
CGI_10003354	Catalase	2.5E-05	-0.76	C	AR
CGI_10017582	Heat shock protein beta-1	1.1E-05	-0.92	AR	AR
CGI_10024262	Transporter	1.6E-05	-0.72	AR	AR
CGI_10003354	Catalase	2.5E-05	-0.76	C	AR
CGI_10017584	Solute carrier family 22 member 13	2.6E-05	-2.26	AR	AR
CGI_10000246	Glutamate carboxypeptidase 2	3.0E-05	-1.02	AR	U2

CGI_10003810	Transporter	5.2E-05	-1.58	AR	AR
CGI_10022548	Solute carrier family 23 member 2	7.2E-05	-0.91	AR	AR
CGI_10001136	N-acetylated-alpha-linked acidic dipeptidase 2	1.1E-04	-0.92	AR	U2
CGI_10018043	Solute carrier family 22 member 4	1.2E-04	-0.88	AR	U1
CGI_10006430	Aminopeptidase N	1.2E-04	-1.37	AR	AR
CGI_10012084	Alpha-crystallin B chain	1.5E-04	-1.01	AR	U2
CGI_10003369	Ammonium transporter Rh type B-A	2.3E-04	-1.36	AR	AR
CGI_10009373	Solute carrier family 43 member 3	3.2E-04	-0.76	AR	AR
CGI_10016164	BTB/POZ domain-containing protein 2	3.3E-04	-0.85	U2	AR
CGI_10024248	Transmembrane protease, serine 9	3.4E-04	-0.73	AR	AR
CGI_10022918	Putative RNA-directed DNA polymerase from transposon BS	4.7E-04	-2.87	AR	AR
CGI_10013419	Calmodulin-like protein	4.9E-04	-0.75	AR	AR
CGI_10021737	Toll-like receptor 2	6.3E-04	-2.51	U2	U1
CGI_10026928	Glutamyl aminopeptidase	6.7E-04	-1.01	U2	C
CGI_10013723	Aquaporin-4	7.0E-04	-1.10	AR	AR
CGI_10014539	Endoplasmic reticulum aminopeptidase 1	7.3E-04	-1.67	U2	U1

Supplementary Table S5. Forward, reverse primers sequences for Real-Time PCR analyzed genes and housekeeping gene.

GeneBank access	Gene name	Forward	Reverse
KX371073	CgClock	5’-TGGGAATGATGTCCAACAGAG-3’	5’-GGTCCATCAATGACAGGAAGT-3’
KT991835	CgCry 1	5’-TCATGAAGCAGCTCAGATACG-3’	5’-ACCTCCCAGTTCAACCAAAG-3’
KX371074	CgCry 2	5’-AACCTTACAGCAAGCACGAA-3’	5’-TGACATCTGGCTGTGGTTTC-3’
KX371075	CgBmal	5’-CACAAGTTCAGGTCAGAGTGTAG-3’	5’-TCACCTGAGGTAGACTGGTTAT-3’
KX371076	CgPeriod	5’-CCGATGACAGAAATCCCAGTAG-3’	5’-CCATCCTATTCTCCTGCTCTTG-3’
(Vogeler et al., 2014)	CgRev-erb	5’-GACTTTGCTGATCGCTTCAAC-3’	5’-CTTTCCAACTGCTCCACATTTC-3’
(Vogeler et al., 2014)	CgRor	5’-CTACGTGAGCAGGTGTTTGA-3’	5’-CGTCCGCTATGTCCTTCAAT-3’
KX371077	CgTim 1	5’-AAAGATCCCGGACACAGTATG-3’	5’-TGGAACTCGTTCCTGACTTG-3’
AB122066	CgEF1	5’-ACCACCCTGGTGAGATCAAG-3’	5’-ACGACGATCGCATTTCTCTT-3’
